# Potential of Hyperthermophilic L-Asparaginase from *Thermococcus sibiricus* to Mitigate Dietary Acrylamide Assessed Using a Simplified Food System

**DOI:** 10.3390/foods14101720

**Published:** 2025-05-12

**Authors:** Maria Dumina, Stanislav Kalinin, Dmitry Zhdanov

**Affiliations:** 1 Federal Research Center “Fundamentals of Biotechnology” of the Russian Academy of Sciences, 117312 Moscow, Russia; 2 Institute of Biomedical Chemistry, 119121 Moscow, Russia

**Keywords:** food-processing contaminants, Maillard reaction, dietary acrylamide, asparagine, food enzyme, hyperthermophilic L-asparaginase

## Abstract

The Maillard reaction is a network of interconnected interactions yielding in formation a number of toxic derivatives in processed foods. Acrylamide, a potential carcinogen and a product of the Maillard reaction, is formed under food processing, predominantly from asparagine and reducing sugars at temperatures over 120 °C. In this study, we investigated the potency of recombinant hyperthermophilic L-asparaginase from *Thermococcus sibiricus* TsAI to mitigate dietary acrylamide by hydrolyzing substrate for its synthesis under various operation conditions. Using a simplified food system for self-cooking, high acrylamide levels were found in baked samples regardless of whether L- or D-enantiomer of asparagine was added. TsAI effectively reduced acrylamide content under various pretreatment conditions, such as temperature, concentration, and time of incubation. The lowest acrylamide level of 1.0–1.1% of the control values or 3.52–3.76 µg/kg was observed in samples pretreated with TsAI 20 U/mL at 90 °C for 20–25 min. Due to the exceptionally high D-asparaginase activity of hyperthermophilic TsAI, the dietary acrylamide content formed from D-asparagine was reduced by 54.8% compared to the control. Comparison of the wild-type TsAI and its mutant reveal that an enzyme displaying enhanced stability is more functional for food-processing application. The native TsAI decreased acrylamide level by 98.9%, while the highly active mutant, with increased structural flexibility, decreased it by only 26.8%. TsAI treatment effectively blocked acrylamide synthesis, but not melanoidin formation via the Maillard reaction, thus not affecting sample characteristics such as color (browning) and aroma, which are important for consumer perception.

## 1. Introduction

Recently, special attention has been paid to safety issues and modern dietary shifts in human nutrition. The quality of products and nutritional culture are primary factors influencing well-being, health, disease risk, and immune function [1].

Food choice depends on many factors, including urbanization, logistics, and food delivery, as well as personal taste preferences and time constraints [2].

The high popularity of snacks, ready-to-eat foods, and progress in food processing have significantly changed the structure of nutrition [3], which is shifting from natural unprocessed products towards processed foods that undergo deep physical and chemical transformations, in particular, during exposure to high temperatures [4].

The fast-growing market of processed foods offers a wide range of processed and ultra-processed products for all ages, such as snacks, cookies, buns, breakfast cereals, and various types of fast food [1]. Toxic derivatives formed during food processing pose serious health risks [5].

One of the major sources of food contaminants formed during food processing is the non-enzymatic Maillard reaction, which is key in food chemistry. The Maillard reaction is a network of interconnected transformations initiated by the interaction between reducing sugars and amino acids under heating above 120 °C [6]. It initiates a cascade of sequential transformations that leads to the formation of Schiff bases, Amadori products, and further rearrangements yielding a wide range of contaminants, some of which are known as toxic substances [3,7].

Acrylamide is an example of a Maillard reaction product that is well-known as a neurotoxin and potential carcinogen [8]. The main route of acrylamide formation in foods via the Maillard pathway starts with interaction between asparagine and sugars [9]. To reduce the formation of dietary acrylamide, two main strategies can be distinguished: eliminating substrates for its synthesis or creating conditions unfavored for the reaction.

The difficulties arise from the complexity of the Maillard reaction effects. Heating and the associated Maillard reaction are the oldest and most widely used food processing technologies to obtain edible products with desired organoleptic properties from raw ingredients. These processes provide protection against microbiological contamination, increasing the shelf life of food. They affect food taste, texture, color, and aroma, playing a huge role in the perception of food [10,11,12]. Both acrylamide-mitigating strategies cannot completely eliminate the risks of impact on these characteristics. Thus, low-temperature processing as well as reducing sugar content can significantly change the organoleptic properties and storage of products.

In this study, as part of a strategy to reduce substrates that “fuel” acrylamide synthesis, we evaluated the ability of archaeal hyperthermophilic L-asparaginase from *Thermococcus sibiricus* TsAI to mitigate dietary acrylamide without affecting other food properties. The enzyme hydrolyzes L-asparagine (L-Asn) to L-aspartic acid and ammonia, thus preventing undesirable interaction between L-asparagine and reducing sugars.

The enzyme was previously expressed in the heterologous host *Escherichia coli*, purified and analyzed [13]. TsAI displayed high activity at the optimum temperature of 90 °C and stability [13], thus the enzyme is considered as promising for food-processing technologies. To evaluate the enzyme potency, a procedure was developed consisting of the following blocs: a simplified food system for self-preparation of samples, thermal processing procedure, and protocols of acrylamide extraction and detection. The efficiency of the wild-type L-asparaginase TsAI was investigated under various operating conditions. Due to convenience, the procedure was used for comparative studies between the wild-type enzyme and its mutant with increased activity and to study some details of the Maillard reaction.

## 2. Materials and Methods

### 2.1. Chemicals

Glucose and fructose (Roquette Freres, Lestrem, France), starch (Pallav Chemicals, Boissard, India), and L-asparagine (Sisco Research Laboratories Pvt. Ltd., Taloha, India) were used to prepare food mixtures according to the basic recipe. D-asparagine (Sisco Research Laboratories Pvt. Ltd., Taloja, India) was used as supplement. Curcumin extract (Bioprex labs, Pune, India) was used as a known food colorant for spectroscopy.

Acrylamide extraction was performed using methanol (Aquametry, Moscow, Russia), potassium ferrocyanide trihydrate (Sisco Research Laboratories Pvt. Ltd., Taloja, India), zinc sulphate heptahydrate (Central Drug House (P) Ltd., Mumbai, India), and acetonitrile (Kriohim, Saint Petersburg, Russia).

Acrylamide (Dia-Gen, Moscow, Russia) was used to prepare a standard solution for HPLC.

All chemicals used in the experiments were of analytical grade.

### 2.2. Enzyme Preparations

Native enzyme preparations of hyperthermophilic L-asparaginase from *Thermococcus sibiricus* (TsAI) were obtained by expression plasmid pET28a-TsAI harboring gene *tsAI*_*mod* (GenBank accession No. MW981255) in heterologous host *Escherichia coli* [13]. For pretreatment of food samples, the enzyme TsAI was purified in our laboratory using ion exchange chromatography according to previously described protocol [13]. The purity of the recombinant enzyme exceeded 98% when assessed by SDS/PAGE electrophoresis. The stock solution of TsAI 689 U/mg (K_m_ 3.8 mM; V_max_ 9.7 mM/min (Supplementary Materials, Figure S1)) at a concentration of 2.84 mg/mL in sodium phosphate buffer (20 mM) containing 1 mM EDTA and 1 mM glycine, pH 7.2–7.3, was used in experimental work.

TsAI mutant form D54G/T56Q was designed, constructed, and expressed in bacterial *E. coli* cells as previously described [14]. The enzyme was purified by ion-exchange chromatography according to described procedure [13]. The stock solution of the mutant TsAI D54G/T56Q 2964 U/mg at a concentration of 3.40 mg/mL was used in experimental work.

The activity of L- asparaginases was determined at the optimal temperature of 90 °C using direct Nesslerization [15].

To determine the static half-life of TsAI and the D54G/T56Q mutant, the enzymes were incubated in optimal buffer (0.05 M Tris-HCl buffer, pH 9.0) at 90 °C without substrate and the activity was assayed every 10 min. The half-life of the enzyme was the time at which the activity decreased by half [16].

### 2.3. Simplified Baking Mixtures

To perform a series of reproducible Maillard reactions in laboratory conditions, a simplified multicomponent system for baking was composed of sugar syrup from equimolar mixture of glucose and fructose 0.80 mM and L-asparagine solution 0.08 mM.

A dough for laboratory testing was prepared in tartlet silicone baking molds (62 × 30 mm) by mixing solutions of L-asparagine, glucose, and fructose. To obtain a homogeneous elastic consistence, starch powder of 1 g per 1 mL was added fractionally at the final step due to swelling behavior of starch.

The number of molds filled with dough depended on the scale of the experiment. The volume of mixed components was 6.67 mL per mold.

In additional experiments, D-asparagine was added to a baking base consisting of sugar syrup and starch instead of L-asparagine at the same content.

### 2.4. Procedure of Enzyme Pretreatment

To prevent the Maillard reaction, L-asparaginase pretreatment of baking mixtures was performed by placing the molds in heating thermostat Julabo WS 100 (Julabo, Seelbach, Germany). Pretreatment was carried out by adding hyperthermophilic TsAI enzyme at a concentration of 1, 5, 10, 15, and 20 U/mL. An equal volume of water was added to the corresponding untreated samples, which served as control. Experiments were performed in the temperature range of 70–95 °C with 5 °C increments and process duration of 5–25 min with 5 min increments.

### 2.5. Thermal Processing of Samples

Thermal processing of the simplified multicomponent system was performed by baking in a laboratory oven at a temperature of 190 °C. The inner dimensions of the baking chamber were as follows: width 520 mm, height 455 mm, depth 340 mm. Duration of the preliminary baking experiment varied from 15 to 60 min. The experimental samples were baked in the middle part of the chamber after visual assessment of the overall appearance: placing the samples in the top oven resulted in quick deep darkening, while samples in the lower part remained pale and undercooked for a long time. The optimal duration of thermal processing in the middle oven was chosen by visual assessment of browning development.

### 2.6. Preparation of Apple Juice Samples

Samples of cloudy apple juice were laboratory prepared from Golden Delicious apples using juicer Philips HR1832/00 (Versuni Home Solutions, Shanghai, China). A total of 4 mL of pressed juice was transferred into 20 mL glass tubes with screw caps. The tubes were incubated at 90 °C for 20 min with or without TsAI addition 20 U/mL. Then, samples were heat-treated by sterilization in autoclave Sanyo MLS-3780 (Sanyo Electric Co., Osaka, Japan) at 121 °C for 20 min. In addition, 1 mL of each sample was taken for acrylamide extraction.

### 2.7. Acrylamide Extraction

The baked samples were cooled at ambient temperature and thoroughly ground using multi-purpose disintegrator Stegler LM-250 (Shenzhen Bestman Instrument Co., Ltd., Shenzhen, China) to obtain a fine powder. The following procedure of acrylamide extraction was performed: 5 mL of methanol was added to 1 g of powder and shaken thoroughly for 3 min. The mixture was centrifuged at 4 °C at 10,000× *g* for 15 min using Cence H1750R (Hunan Xiang Yi Laboratory Instrument Development Co., Ltd., Changsha, China). Then, 3 mL of the clear supernatant was transferred to a 15 mL plastic tube and mixed with 0.1 mL of Carrez I (K_4_[Fe(CN)_6_] × 3H_2_O) and 0.1 mL Carrez II (ZnSO_4_ × 7H_2_O) solutions. After shaking for 30 sec, the mixture was stored at room temperature for 20 min and then centrifugated (4 °C, 10,000× *g*, 15 min). A total of 2.5 mL of clear supernatant of each sample was transferred to a glass tube and evaporated at 60 °C. The acrylamide residue was dissolved in 1 mL of acetonitrile:water (1:1) and filtered through a 0.22 μm filter.

### 2.8. HPLC Analysis

Analysis of acrylamide content in baked samples was carried out using high performance liquid chromatography (HPLC). Quantification was performed with Agilent 6130 chromatography-mass spectrometer (Agilent Santa Clara, CA, USA) equipped with autosampler Spark Holland Midas (Spark Holland, Emmen, Netherlands), pump Knauer Smartline S1000 (Knauer, Berlin, Germany) with a degasser. Vials containing the samples were loaded into an autosampler carousel and 20 μL of each sample was injected into a Phenomenex Onyx C18 column (Phenomenex, Torrance, CA, USA) (50 cm × 4.6 mm). The composition of the liquid phase was as follows: 20 mM ammonium formate and 0.1% formic acid in aqueous solution and acetonitrile in a ratio of 50:50 (*v*/*v*) at a flow rate of 0.6 mL/min.

For acrylamide detection, parameters were set as follows: gas flow (nitrogen) 11 L/min, gas temperature 320 °C, capillary voltage of 3000 V, desolvation voltage (Fragmenter) 80 V. The protonated molecular ion [M+H]+ of acrylamide was detected at a mass-to-charge ratio ratio m/z 72.

### 2.9. Sensory Analysis

Sensory evaluation involved 21 untrained individuals—11 men and 10 women—aged 21 to 56 years. Freshly baked samples, untreated and treated with TsAI 20 U/mL, were evaluated by staff of the Research Center within 2.5 h. Participants were asked to assess color, aroma, texture, hardness, volume, porosity, and overall appearance of samples prepared using 15 and 20 min pretreatment regimens. The 9-point hedonic scale ranging from 9 (like extremely) to 1 (dislike extremely) was used for evaluation [17].

### 2.10. Evaluation of Color Differences

Absorbance spectra of baked samples pretreated for 15 and 20 min with or without TsAI addition 20 U/mL and curcumin, a yellowish-orange food colorant, were recorded using EzDrop 1000C spectrophotometer (Blue-Ray Biotech, New Taipei, Taiwan) in the range of 190 nm to 1000 nm at a bandwidth of 1 nm (Supplementary Materials, Figure S2). The data were converted to reflectance spectra covering the visible wavelengths region from 380 to 780 nm. Analysis was performed in Color Calc. software (version 2022) by employing the CIE color-difference evaluation system: L* (perceptual lightness), a* (green–red), and b* (blue–yellow) [18,19]. The calculations of the color difference (color change, ΔE) were made as described previously [20,21]. The ratios between the calculated values ΔE and the tolerance of the human eye to color differences were interpreted as follows: ΔE ≤ 1—color difference is barely perceptible to the naked eye, 1 < ΔE ≤ 2—color difference can be detected only under careful comparison, 2 < ΔE ≤ 3—slightly visible differences, 3 < ΔE ≤ 5—visible differences, ΔE > 5—significant color differences, easily noticeable to the naked eye, observer notices two different colors.

### 2.11. Statistical Analysis and Software Programs

All experimental results were expressed as mean value ± standard error calculated from three parallel experiments. Statistical analysis and data visualization were performed by one-way analysis of variance (ANOVA), two-way analysis of variance (two-way ANOVA) using Microsoft Excel (version 2010) and software GraphPad Prism 9.5.1.733, NCSS (Number Cruncher Statistical System) 2023 version 23.0.2.

## 3. Results and Discussion

### 3.1. Procedure for Dietary Acrylamide Formation and Detection in Thermal-Processed Food Samples

Investigation of factors influencing or affecting acrylamide formation in foods requires self-cooking of food samples. In laboratory conditions, a food system with good reproducibility is highly desirable for multiple repeats, including comparative. To investigate the effects of L-asparaginase pretreatment on acrylamide mitigation under different operation parameters, we used a simplified food mixture for baking, composed of L-asparagine, glucose, and fructose as acrylamide precursors, and starch as a chemically defined flour substitute.

**Figure 1:**
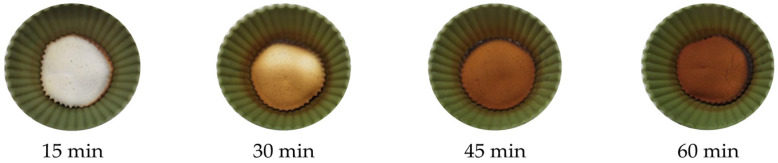
Typical appearance of samples baked at 190 °C for 15–60 min.

The thermal processing regime was chosen in an additional experiment. At a baking temperature of 190 °C, the results of the treatment were highly dependent on the laboratory oven used, the volume of the dough in silicone molds, and the position of the forms in the oven volume. By placing molds in the middle part of the oven and varying time of heat treatment from 15 to 60 min, a duration of 30 min was found to be optimal to obtain both the characteristic color of the baked samples and a fine powder for further acrylamide extraction (Figure 1).

HPLC detection of dissolved acrylamide residues reveals an increase in acrylamide levels from 164.02 ± 26.55 µg/kg (15 min) to 457.65 ± 48.45 µg/kg (60 min); 30 min baking of the simplified food system resulted in the formation of acrylamide 342.36 ± 46.31 µg/kg.

### 3.2. Mitigation of Dietary Acrylamide in Simplified Food System by Pretreatment with L-Asparaginase TsAI

#### 3.2.1. Overall Procedure of Acrylamide Detection in Baked Samples Pretreated with Hyperthermophilic L-Asparaginase TsAI

To prevent the Maillard reaction, L-asparaginase pretreatment should be applied before the main thermal processing of foods to eliminate L-asparagine, which serves as a substrate for acrylamide synthesis. The efficiency of TsAI in depleting L-asparagine in a multicomponent food mixture depends on a combination of factors, including pretreatment temperature, enzyme concentration, incubation time, and enzyme stability under given conditions.

**Figure 2:**
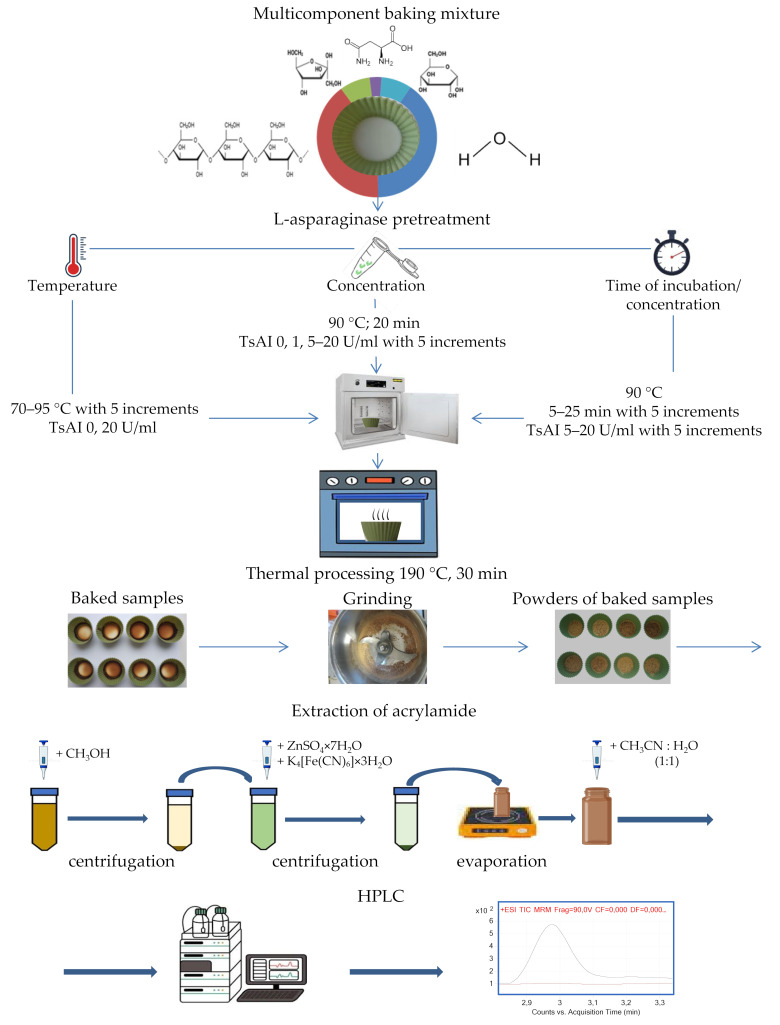
Overall scheme of investigation of L-asparaginase TsAI activity in reducing dietary acrylamide depending on temperature, concentration, incubation time.

To evaluate TsAI potential in reducing acrylamide content under various treatment conditions, experimental work was carried out according to the scheme presented in the Figure 2, which includes the above-described procedures for preparing food mixtures, thermal processing, extraction, and acrylamide detection.

#### 3.2.2. Sensory and Color Characteristics of Baked Samples

In total, more than 100 samples were pretreated with TsAI under various conditions, baked, and analyzed. When analyzing the mechanical characteristics of the baked samples, no significant differences were observed between control and TsAI-pretreated samples in any of parameters. Volume, texture, porosity, and hardness were practically identical regardless of operating conditions tested. Surprisingly, TsAI treatment did not affect color and aroma of the samples.

**Figure 3:**
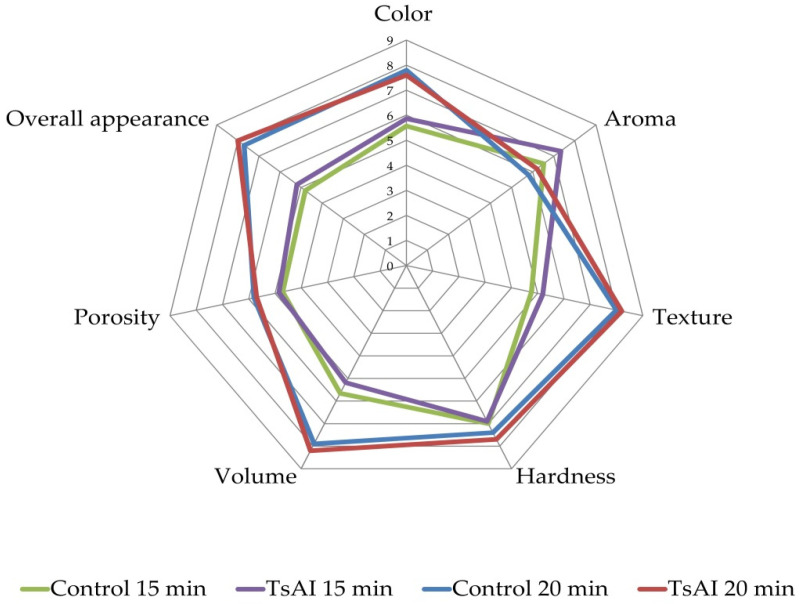
Sensory profile of TsAI-untreated (control) and TsAI-treated samples after 15 min and 20 min preincubation.

Our observations were confirmed by the results of sensory evaluation. Untrained participants representing typical consumers assessed characteristics of two groups of baked samples: 15 min pretreatment—control and TsAI-treated, 20 min pretreatment—control and TsAI-treated. Participants showed a greater preference for samples prepared with the 20 min pretreatment regimen, so a number of characteristics differed significantly between groups (Figure 3). Minor differences between enzyme-treated and untreated (control) samples within groups were not statistically significant (Table 1). The exception was the aroma for 15 min pretreatment samples: the TsAI-treated samples were significantly more preferable.

In addition, color differences were analyzed using UV–visible spectroscopy. Visible light spectra obtained from the same samples used for sensory evaluation and curcumin, a natural yellow to orange food colorant, were recorded and converted into CIE color values (Table 2). Samples of both groups displayed similar CIE values. Within the group, overall color differences (ΔE) of samples preincubated for 15 or 20 min with or without (control) TsAI were estimated to be 3.62 and 1.42, respectively. The value of 3.62 corresponds to visible differences; however, in view of typical heterogeneity in the overall color of baked products, they can be considered as acceptably close and not significant for such products. The color differences between TsAI-treated and untreated samples after 20 min incubation, as assessed by ΔE, were minor, detectable only by careful comparison, and not significant.

**Figure 4:**
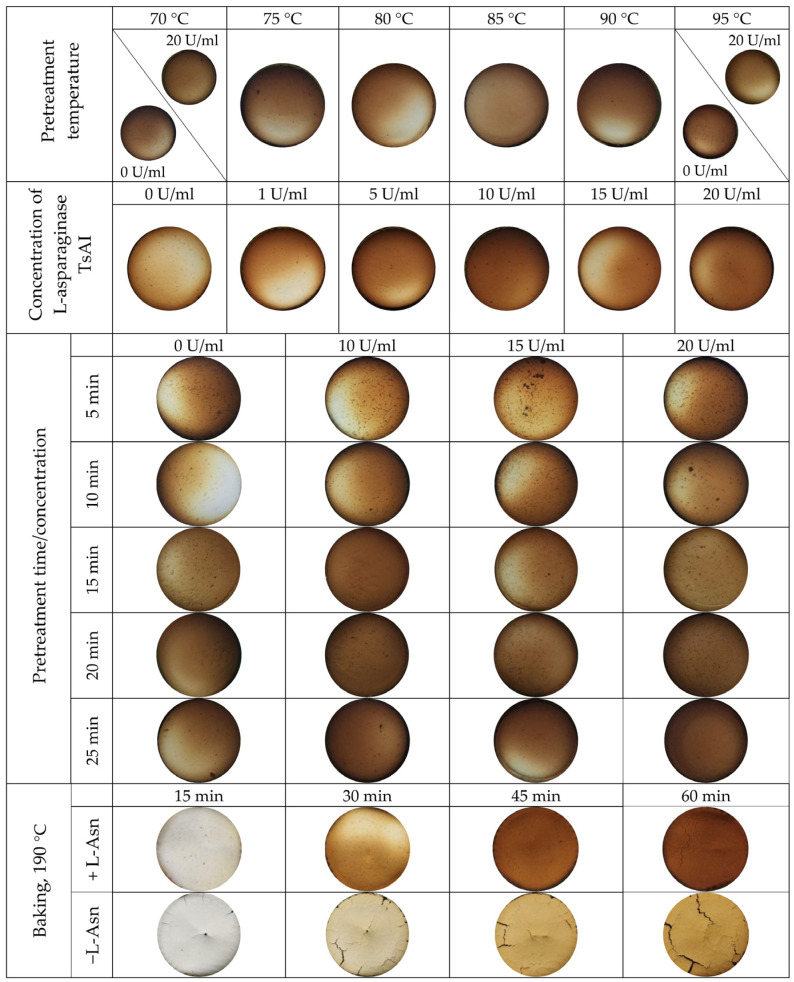
Typical appearance of baked samples pretreated with TsAI under various conditions. Typical appearance of samples with or without adding L-asparagine (L-asn), +L-asn and −L-asn, respectively, baked at 190 °C for 15–60 min.

Thus, TsAI treatment did not affect sensory characteristics and color of the samples. These characteristics are one of the key factors that govern consumer behavior. Visual and olfactory perception of food is the first step of food quality assessment. On the other hand, it is known that the browning of bakery products and their flavor are also correlated with the intensity of the Maillard reaction [10], due to acrylamide formation. In this study, we can state that TsAI treatment did not impact color intensity, aroma, and overall appearance of baked samples (Figure 3 and Figure 4, Table 1 and Table 2). Nevertheless, simply excluding asparagine as a “fuel” for acrylamide formation from composition significantly decreased browning of samples (Figure 4).

#### 3.2.3. Acrylamide Content in Baked Samples Depending on TsAI Pretreatment Temperature

Effective hydrolysis of L-asparagine by TsAI in a multicomponent food mixture depends on both temperature and stability of enzymes under certain conditions. In our previous study, TsAI was active at the temperature range of 70–95 °C [13]. The enzyme retained more than 85% of its initial activity towards L-asparagine when incubated for 20 min at these temperatures and optimum pH (0.05 M Tris-HCl buffer, pH 9.0) [13].

**Figure 5:**
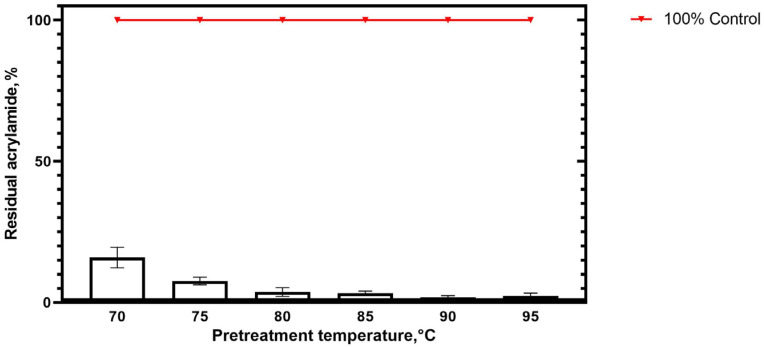
Relative residual acrylamide content in samples pretreated with TsAI 20 U/mL at different temperatures for 20 min.

In a preliminary experiment using a simplified food system to prevent the Maillard reaction, baking mixtures were pretreated with TsAI at a concentration of 20 U/mL and heating at temperatures of 70–95 °C with 5 °C increments. Enzyme activity in acrylamide reduction was assessed after enzyme pretreatment for 20 min and subsequent baking for 30 min at a temperature of 190 °C (Figure 2, Figure 4 and Figure 5).

Acrylamide content was reduced in all samples pretreated with TsAI 20 U/mL at all temperatures tested compared to the respective controls (Figure 5). At 70 °C, residual acrylamide was estimated at 15.94% of the control value. The lowest residual acrylamide content was observed when TsAI pretreatment was carried out at 90 °C and 95 °C—1.76% and 2.33%, respectively, compared to the controls.

The results obtained for TsAI in the presence of a mixture of food ingredients, in general, were consistent with its biochemical properties observed in buffer at optimal pH value [13]. Displaying the highest activity in L-asparagine hydrolysis at 90 °C [13], the hyperthermophilic L-ASNase TsAI was stable in the presence of food ingredients and reduced acrylamide content by about 98% in baked samples pretreated at 90 °C.

When samples were mixed from room temperature components and pretreated at 95 °C, a gradual increase in internal temperature occurred depending on thermal conductivity of viscous food composition. When incubated at 95 °C, the mixture passed through the optimum TsAI temperature of 90 °C, which contributed to a more efficient acrylamide reduction than pretreatment at temperatures below 90 °C. In addition, with heating to temperatures above the optimum, at which the enzyme remains stable, the reaction rate increases. At 95 degrees, the reduction of acrylamide was more than 97% of the control value (Figure 5).

#### 3.2.4. Effective TsAI Concentrations for Acrylamide Reduction

**Figure 6:**
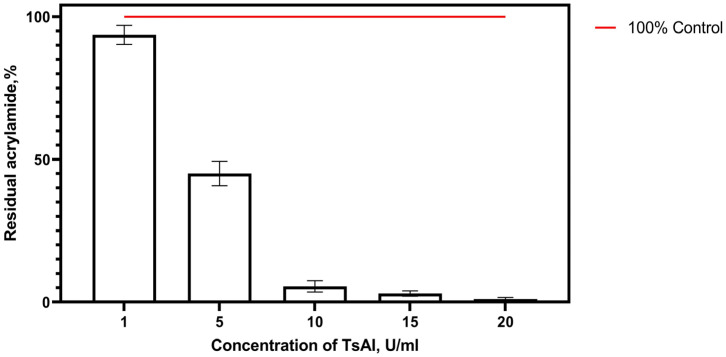
Relative residual acrylamide content in samples pretreated with TsAI at various concentrations for 20 min.

In an attempt to estimate TsAI working concentrations efficient enough to decrease acrylamide levels in foods, TsAI was added to samples at concentrations of 1, 5, 10, 15, and 20 U/mL. According to the experimental data, no significant differences in acrylamide content were observed between control samples and samples pretreated with TsAI at a concentration of 1 U/mL. A two-fold decrease in acrylamide level occurred when samples were pretreated with TsAI at a dosage of 5 U/mL. The residual acrylamide content gradually decreased to 5.49%, 3.01%, and 1.10% relative to control values with an increase in TsAI concentration to 10, 15, and 20 U/mL, respectively (Figure 6).

#### 3.2.5. Determination of Effective Time/Concentration Ratio of TsAI for Acrylamide Reduction

The effect of TsAI concentration and incubation time on the content of acrylamide at the pretreatment step was investigated. The dynamics of acrylamide reduction treated with TsAI at concentrations of 10, 15, and 20 U/mL was recorded at time points from 5 to 25 min. Further increase in incubation at 90 °C led to excessive moisture loss and samples drying.

For different combinations of concentration/incubation time, residual acrylamide levels varied from 1.03% to 97.18% of the corresponding control values. TsAI concentration and time of preincubation, as well as their combination, had a significant effect (*p* < 0.0001) on residual acrylamide levels (Table 3).

**Figure 7:**
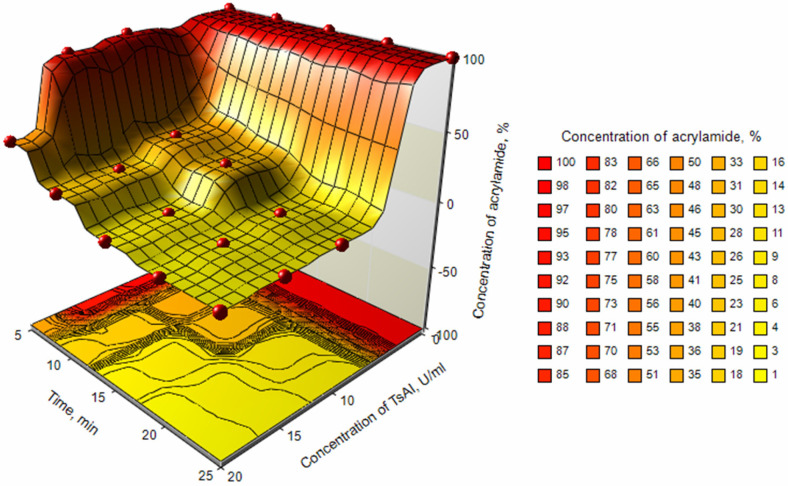
Surface plot of relative residual acrylamide depending on TsAI dosage–incubation time.

Based on time-dependent acrylamide reduction, three variants of preincubation procedure can be distinguished (Figure 7).

For short 5 min pretreatment, a high concentration of TsAI~18–20 U/mL is required (Figure 7). A fast two-fold decrease in acrylamide level compared to the control was observed for TsAI 20 U/mL, while lower TsAI concentrations were ineffective for a short 5 min treatment. Thus, samples pretreated with TsAI at a dosage of 10, 15, and 20 U/mL reduced acrylamide content by 2.82, 7.51, and 57.75% compared to control values, respectively, during 5 min incubation.

Increasing the pretreatment time to 10–15 min eliminated strong differences between TsAI treatment at a concentration of 15 and 20 U/mL and resulted in a decrease in acrylamide content by ~78–79% at 10 min and 93–94% at 15 min compared to the control.

For prolonged 20–25 min incubation, a significant reduction in acrylamide level was revealed for all TsAI concentrations tested (Figure 7). The lowest acrylamide content—1.0–1.1% of the control value or 3.52–3.76 µg/kg—was observed in samples incubated with TsAI 20 U/mL for 20–25 min (Figure 7).

### 3.3. Comparative Study of TsAI Wild-Type and Its Highly Active Mutant to Mitigate Dietary Acrylamide

The simplified food system is a convenient tool for comparative studies, in particular of agents that reduce formation of toxic derivatives of the Maillard reaction.

Using the simplified food system, the effect of engineered form of the hyperthermophilic L-asparaginase TsAI was evaluated.

Previously, a mutant form of TsAI—TsAI D54G/T56Q—was obtained using protein engineering [14]. Two substitutions in close proximity to the enzyme–substrate interaction site affected both the specific activity and flexibility of enzyme structure. The specific activity of D54G/T56Q double mutant increased more than two-fold compared to parent TsAI at the optimum temperature of 90 °C—5038 U/mg vs. 2066 U/mg, respectively [14]. However, increased conformational flexibility of hyperthermophilic TsAI D54G/T56Q was accompanied by a decrease in stability. TsAI-D54G/T56Q displayed more narrow temperature-dependent activity profile compared to native TsAI [14].

In this study, for food industry application, the half-life of the mutant enzyme was assayed at optimum temperature and pH. The half-life of D54G/T56Q mutant was estimated to be 35 min at 90 °C, and 69 min for the TsAI wild-type, and both values seemed sufficient for efficient acrylamide reduction.

Evaluation of the efficiency of D54G/T56Q mutant 20 U/mL in mitigating dietary acrylamide at 90 °C and preincubation time of 20 min revealed unexpected results. The native TsAI enzyme decreased acrylamide level by 98.9%, while the mutant form reduced its content by only 26.8% compared to the control. The results confirm the complexity of enzyme behavior under real conditions. The stable wild-type TsAI with a lower specific activity (under optimal conditions) was more functional in mitigating acrylamide than the highly active mutant with impaired stability.

Recent structural data confirm that the mutated residues increased the flexibility of the structure around the active site, facilitating reorientation and catalysis. At the same time, the hydrogen bond network important for thermostability was disrupted. In the food system, the loss of mutant activity had a critical impact on acrylamide mitigation.

### 3.4. D-Enantiomer of Asparagine in Food: Acrylamide Formation and Stereospecificity of TsAI Catalytic Reaction

Along with L-enantiomers, food products contain D-enantiomers of amino acids. In particular, D-asparagine is found in measurable quantities in many products, such as tomato, carrot, garlic, apple, and grapefruit [22,23]. Except raw vegetables and fruits, D-asparagine can accumulate in food due to racemization during storage, microbial fermentation, and food processing [22,24].

**Figure 8:**
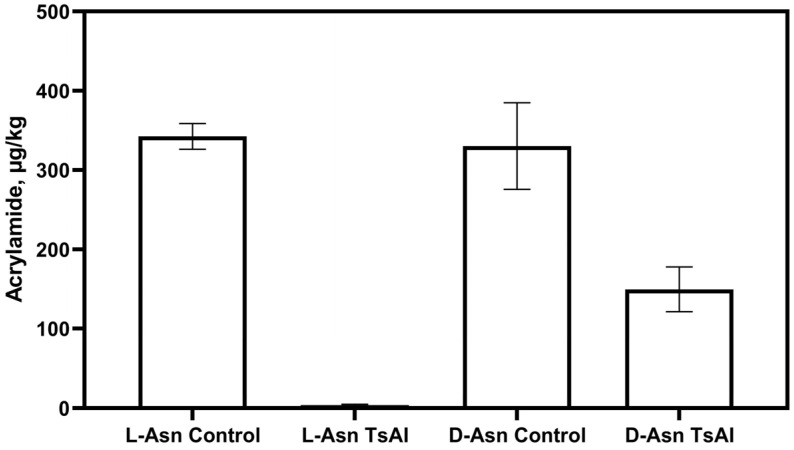
Residual acrylamide levels in baked samples, containing L-asparagine (L-asn) or D-asparagine (D-asn), untreated (control) or pretreated with TsAI 20 U/mL for 20 min.

To investigate the efficiency of conversion D-enantiomer of asparagine to acrylamide in the Maillard reaction, L-asparagine was substituted with D-enantiomer in the simplified system. The samples were baked at 190 °C during 30 min. Analysis of extracted acrylamide reveals that its amounts formed from L-asparagine and D-asparagine were practically equal (Figure 8).

The Maillard reaction is accompanied by two interrelated processes: enolization and racemization of sugars via the Lobry de Bruyn–Alberda van Ekenstein transformation and racemization of amino acids from Amadori compounds, which complicate the Maillard reaction network [24,25]. It has been previously shown that the use of different enantiomers can influence the formation of melanoidins as a result of the Maillard reaction [24]. In this study, substitution of L-asparagine with D-enantiomer did not affect either formation or content of the low-molecular-weight product of the Maillard reaction—acrylamide.

L-asparaginases of different origin are characterized by different abilities to catalyze conversion of structurally related substrates; in particular, a number of enzymes exhibit D-asparaginase co-activity [26,27]. The activity of mesophilic L-ASNases towards D-asparagine is 1–6% of the activity when L-asparagine is used as a substrate [28,29].

In this study, the hyperthermophilic TsAI asparaginase exhibited an extraordinarily high relative activity towards D-asparagine, measured by direct Nesslerization—62% of the L-asparaginase activity. The results were confirmed in real conditions using a multicomponent food mixture containing D-asparagine instead of the L-enantiomer. Preincubation of baked samples with TsAI at a concentration of 20 U/mL at 90 °C for 20 min reduced acrylamide content by 54.8% when D-asparagine was added, and by 98.9% when L-asparagine was used as a substrate for acrylamide synthesis (Figure 8).

The results obtained using a simplified food system indicate a high activity of hyperthermophilic L-asparaginase TsAI in reducing acrylamide levels, as well as efficiency in hydrolyzing a mixture of L- and D-asparagine, which are substrates for the non-enzymatic synthesis of toxic derivatives of the Maillard reaction.

### 3.5. Reducing Acrylamide Level in Thermally Processed Apple Juice by TsAI Pretreatment

In addition to baked and fried foods, high levels of acrylamide were found in various types of beverages, such as dark beer [30], coffee [31], and even juice [32]. For example, prune juice was reported to contain~138–268 µg/kg acrylamide, which is comparable to the level in French fries [32]. In juice, toxic Maillard reaction products are formed during heat treatment and storage [32,33,34].

To evaluate TsAI activity in mitigating acrylamide content in real food, experiments were performed using cloudy apple juice. Based on the results in the simplified food system, samples of freshly pressed cloudy apple juice were incubated with and without TsAI 20 U/mL at 90 °C for 20 min, heat treated by sterilization, and analyzed. Sterilization was shown to induce acrylamide formation at a level of 173.49 ± 34.76 µg/kg in untreated apple juice samples, while acrylamide was reduced to undetectable levels in all samples treated with TsAI 20 U/mL.

Studies confirm that sterilization can cause acrylamide formation in foods [35,36,37]. However, thermal sterilization is one of the most widely used processing methods for food preservation. Modifications in the thermal sterilization regime can alter sensory profiles of products [38].

TsAI displayed high activity in a natural food raw material of multicomponent composition. In a homogeneous liquid system of sterilized cloudy apple juice, acrylamide content was reduced by TsAI to undetectable levels.

It was also reported that acrylamide formation can occur at temperatures below 100 °C [32]. Conversion of asparagine to acrylamide was observed in unusual reaction conditions, for example, under long-term storage of apple juice [34]. Hydrolysis of asparagine by treatment with L-asparaginase in such food products can also prevent acrylamide formation during storage.

Food-processed contaminants, in particular, acrylamide formed from asparagine and sugars in the Maillard reaction, act as “silent killers”, causing systemic effects with prolonged exposure even in minor amounts. Currently, various approaches are proposed to reduce their content in food products. A brief summary of techniques used to mitigate dietary acrylamide in bakery products is provided in Table 4.

Treatment with acrylamide-reducing enzymes during high-temperature food-processing is one of the most effective and promising approaches (Table 4). Hyperthermophilic enzymes can be considered as the most prospective for the vacant niche in high-temperature food technologies. In this study, TsAI decreased acrylamide levels in the simplified food system up to 98–99% without significantly affecting aroma or color, both important aspects for consumers, thus confirming the melanoidins formation via the Maillard pathway. High efficiency of the enzyme was confirmed in sterilized apple juice.

According to available data, L-asparaginase from *Thermococcus zilligii* reduced acrylamide content by 80% in French fries [51]. The reduction in acrylamide content by L-asparaginases from mesophilic fungi *Aspergillus* sp. was as follows: model system—90% [52], bread—57% [53], biscuits—69% [54], and potato chips—60% [55].

Currently, two commercially available L-asparaginases from the mesophilic fungi *Aspergillus* sp. have been approved for food industry application: Acrylaway^®^ (Novozymes A/S, Bagsvaerd, Denmark) from *Aspergillus oryzae* and PreventASe^TM^ (DSM, Heerlen, The Netherlands) from *Aspergillus niger* [56].

In high-temperature food technologies, L-asparaginases act on asparagine content before high-temperature heating [57]. When the temperature increases to 120 °C and above, L-asparaginase preparation is inactivated and irreversibly denatured. Under dietary expose, the denatured enzyme does not exhibit activity, and no signs of toxicity have been reported [57,58]. Based on the safety assessment, L-asparaginase preparations have been recognized as generally recognized as safe (“GRAS”) by the United States government and approved as food additives by the World Health Organization [57,58].

Due to substrate specificity, L-asparaginase depletes asparagine and, to a minor extent, glutamine, without affecting the levels of other amino acids. Asparagine (DL) is hydrolyzed by the enzyme to aspartic acid (DL) and ammonia.

Aspartate (DL) is one of the predominant amino acids (among the corresponding amino acid enantiomers) naturally present in raw and processed foods, a macronutrient [22,23,59]. L-aspartate is a normal component of proteins.

Ammonia and its compounds are normally present in foods and are not considered to pose a health risk [60]. Ammonia and its ions are recognized to be integral components of normal metabolic processes. Ammonium salts are widely used in the food industry [60].

The total human exposure to aspartate (DL) and ammonia through diet is several orders of magnitude greater than the estimated exposure from L-asparaginase-mediated hydrolysis of asparagine (DL).

In addition to safety issues, it is necessary to note the limitations of L-asparaginase application in the food industry. L-asparaginases are biocatalysts. The efficiency of this strategy for acrylamide mitigation is strongly dependent on enzyme stability, even for thermophilic forms. For high efficiency of L-asparaginase treatment, homogeneous systems of moderate viscosity are preferred, which can provide better distribution and access for efficient hydrolysis of asparagine during a relatively short pretreatment period.

Asparagine is the main substrate for acrylamide synthesis, however, alternative reactions of acrylamide formation are reported [61]. Due to the selectivity of L-asparaginases towards asparagine, enzymatic treatment is unable to block other pathways responsible for acrylamide synthesis in foods. However, such food examples are limited [37,61,62].

Currently, L-asparaginase treatment is one of the most effective strategies to reduce/block acrylamide formation in different types of thermally processed foods of various composition. However, the following problems/issues need to be solved for wide application of asparaginases in food-processed technologies: availability, stability, high dependence of the effect on the type of food, treatment conditions, and enzyme concentration. Solving the first two problems, expression of stable L-asparaginase from hyperthermophilic archaea originating from harsh environments was performed after codon optimization in fast-growing bacteria *E. coli* [13]. In this study, evaluation of TsAI activity in dietary acrylamide reduction in a simplified food system at various dosages and treatment conditions will contribute towards the next step, which is its real application in food processing.

## 4. Conclusions

In attempts to investigate the potential of a novel enzyme in high-temperature food processes, a procedure was developed consisting of the following parts: a simplified food system of defined composition for multiple self-cooking of samples; a baking procedure as a type of thermal processing; and protocols of acrylamide extraction and detection to evaluate the effectiveness of enzyme pretreatment. The procedure turned out to be universal and convenient for comparative studies and investigating factors affecting formation of food-processed contaminants.

The recombinant hyperthermophilic asparaginase TsAI effectively reduced acrylamide content in a simplified food system, by hydrolyzing L-asparagine along with its D-enantiomer, which are substrates for acrylamide synthesis in the Maillard reaction. The lowest acrylamide level of 1.0–1.1% of the control values was observed in baked samples pretreated with TsAI at the following parameters: enzyme dosage—20 U/mL, treatment temperature—90 °C, incubation time—20–25 min. The obtained results are the starting point for further enzyme application for pretreatment of various foods subjected to different thermal processing. This requires large quantities of the enzyme.

The problem of the availability of thermostable L-asparaginase can be solved by scaling up laboratory techniques, optimization of fermentation in bioreactors with a focus on critical process parameters [63,64,65]. Currently, industrial production of native *E. coli* L-asparaginase EcAII worldwide meets the growing demand for the enzyme for biomedical applications. The high level of TsAI expression at laboratory scale [13] in the same bacterial host *E. coli* provides hope for scaling up techniques based on the success of industrial EcAII production for biomedicine.

## Figures and Tables

**Table 1:** Sensory evaluation of TsAI-untreated (control) and TsAI-treated samples.

Sample	Color	Aroma	Texture	Hardness	Volume	Porosity	Overall Appearance
15 Min Pretreatment							
**Control**	5.57 ± 1.18	6.52 ± 2.15	4.76 ± 1.65	7.00 ± 1.77	5.66 ± 1.50	4.71 ± 1.71	4.80 ± 1.33
**TsAI**	5.86 ± 1.33	7.33 ± 2.08	5.19 ± 1.74	6.90 ± 1.90	5.19 ± 1.22	4.85 ± 1.70	5.19 ± 1.45
***p***	>0.05	<0.05	>0.05	>0.05	>0.05	>0.05	>0.05
**20 Min Pretreatment**							
**Control**	7.80 ± 1.48	5.80 ± 2.49	7.95 ± 1.39	7.42 ± 2.98	7.90 ± 1.55	5.81 ± 2.11	7.71 ± 1.41
**TsAI**	7.57 ± 1.65	6.19 ± 2.33	8.23 ± 1.09	7.71 ± 2.87	8.23 ± 1.65	5.71 ± 2.51	8.04 ± 1.45
***p***	> 0.05	>0.05	>0.05	>0.05	>0.05	>0.05	>0.05

**Table 2:** CIE colorimetric values.

Sample	L*	a*	b*	ΔE
**Curcumin**	71.53	−14.36	72.89	
**15 Min Pretreatment**				
**Control**	63.15	5.34	61.97	3.62
**TsAI**	66.00	1.05	62.09	
**20 Min Pretreatment**				
**Control**	64.83	7.39	67.40	1.42
**TsAI**	65.04	5.09	65.98	

**Table 3:** Two-way ANOVA analysis for residual acrylamide.

Independent Parameters	Range	ANOVA for RAA (Response)					
		SS	DF	MS	F Value	*p*	Significance
Concentration	10–20 U/mL	66,767.46	3	22,255.82	43,667.95	<0.0001	Significant
Incubation	5–25 min	25,541.42	4	6385.35	12,528.65	<0.0001	Significant
Concentration/incubation	10–20 U/mL/ 5–25 min	12,269.75	12	1022.48	2006.19	<0.0001	Significant

RAA—residual acrylamide, SS—sum-of-squares, DF—degrees of freedom, MS—mean square.

**Table 4:** Summary of approaches to mitigate dietary acrylamide.

Method		Advantages	Disadvantages	Example		Reference
				Food Matrix	Reduction in AA ^1^, %	
**Physical**	Microwaves	Simplicity in technological application	Controversial results	Biscuit	30.9	[39]
	Reduction in heat treatment	Ease of application	Impact on organoleptic characteristics	Biscuit	60.0	[40]
	Vacuum	Reducing heat treatment duration	Affect product texture	Cookies	53.0	[41]
**Chemical**	CaCl_2_	Precise regulation of concentration and composition of chemical components	Influence on the organoleptic characteristics	Cookies	58.0	[42]
	Pectin			Biscuit	67.0	[43]
	Tartaric acid			Biscuit	52.0	[43]
	Replacing ammonium bicarbonate			Biscuit	87.2	[44]
**Biological**	**Fermentation**					
	Lactic acid bacteria	Ease of use, availability of components	Strict conditions and process control for desirable effects	Wheat bread	48.7	[45]
	Baker’s yeast			Sourdough bread	40.1	[45]
				Biscuit	75.2	[46]
	**Herbal Ingredients**					
	Green tea extract	Natural components	Unstable effects	Biscuit	73.3	[46]
	Ground ginger			Biscuit	23.7	[47]
	Antioxidants of bamboo leaves			Cookie	63.9	[48]
	Tea polyphenols			Cookie	43.0	[48]
	Vitamin E			Cookie	71.2	[48]
	**Enzymatic**					
	Glucose oxidase	High efficiency	High cost	Biscuit	63.9	[46]
	Acrylamide amidohydrolase			Cookie	95.0	[49]
	Asparaginase			Cookie	54.0	[50]
				Cracker	80.0	[50]
				Biscuit	96.0	[50]

^1^ AA—acrylamide.

## Data Availability

The original contributions presented in the study are included in the article/Supplementary Materials, further inquiries can be directed to the corresponding author.

## Supplementary Materials

The following supporting information can be downloaded at: https://www.mdpi.com/article/10.3390/foods14101720/s1. Figure S1: Graphs of kinetic curves for determining the kinetic parameters of TsAI L-asparaginase; Figure S2: UV–vis spectra of curcumin, TsAI-untreated (control), and TsAI-treated (TsAI) samples; Figure S3: LC–MS/MS chromatogram of acrylamide content in samples containing L-asparagine; Figure S4: LC–MS/MS chromatogram of acrylamide content in samples containing D-asparagine.

## Abbreviations

The following abbreviations are used in this manuscript:

AA	Acrylamide
RAA	Residual acrylamide
