# Supplementary material for: Potential of Hyperthermophilic L-Asparaginase from Thermococcus sibiricus to Mitigate Dietary Acrylamide Assessed Using a Simplified Food System
Source: Foods. 2025 May 12;14(10):1720. doi: 10.3390/foods14101720 (PMC12111007; doi:10.3390/foods14101720)
Supplement: Supplementary file 1 [file foods-14-01720-s001.zip › foods-3604236-supplementary.pdf]

Supplementary Materials

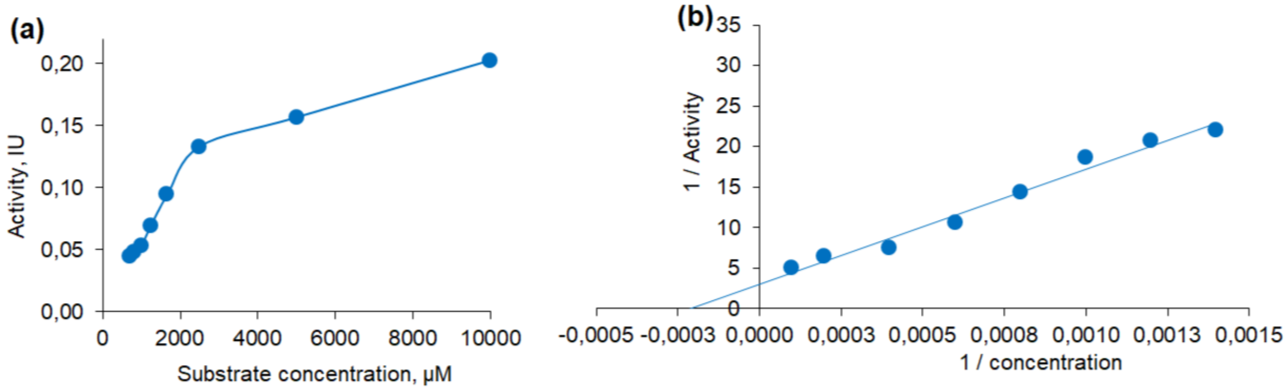

Figure S1. Graphs of kinetic curves for determining the kinetic parameters of TsAI L-asparaginase: **(a)** the graph of the dependence of enzymatic activity on L-asparagine concentration; **(b)** double-reciprocal Lineweaver-Burk plot.

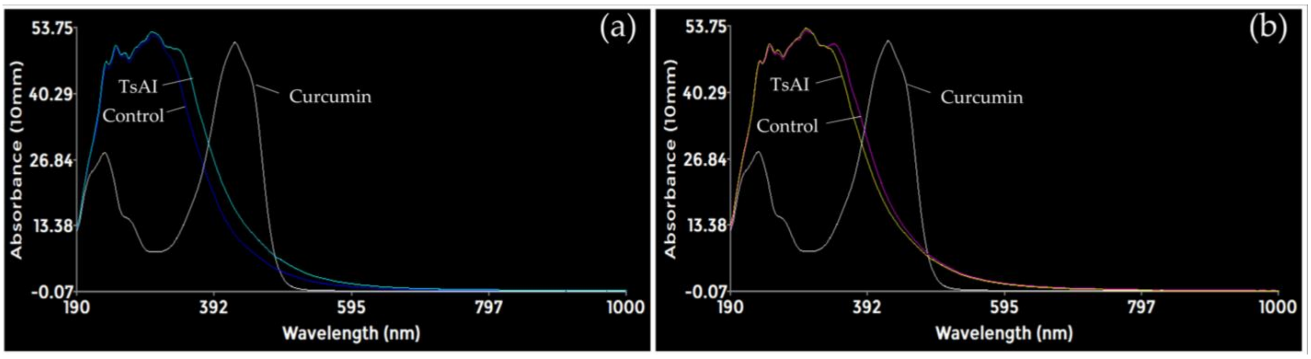

Figure S2. UV-Vis spectra of curcumin, TsAI-untreated (control) and TsAI-treated (TsAI) samples **(a)** after 15 minutes of incubation, **(b)** after 20 minutes of incubation.

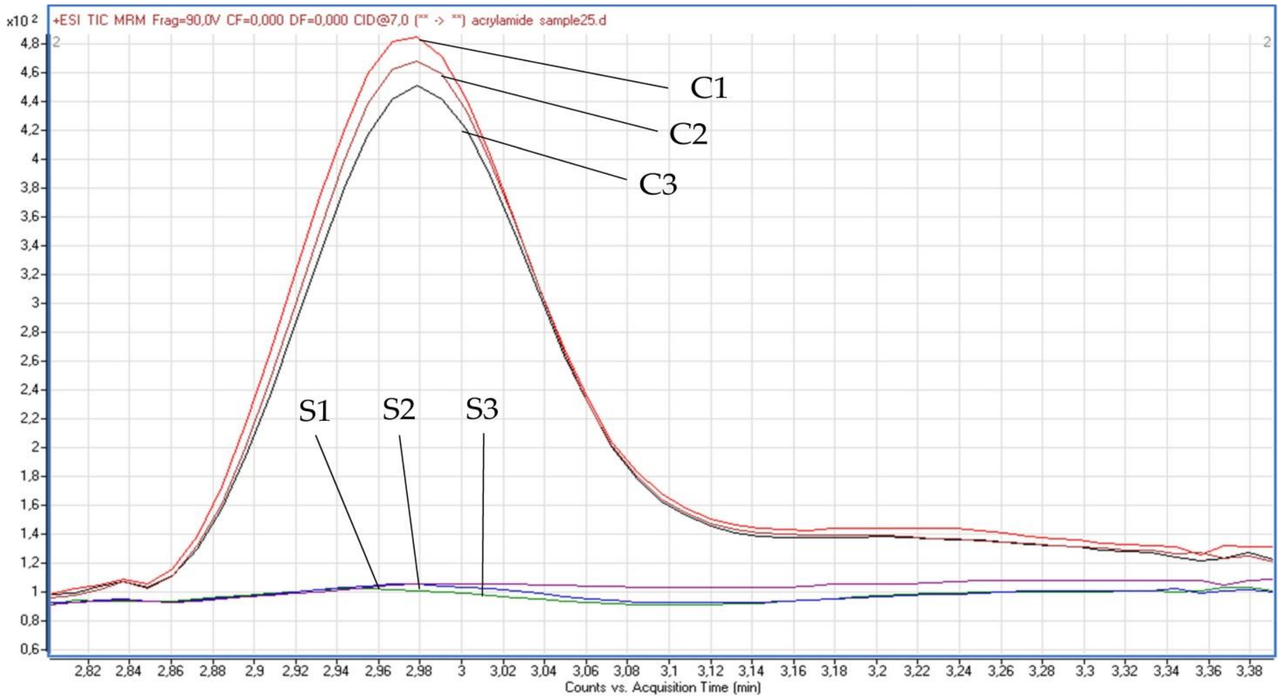

Figure S3. LC-MS/MS-chromatogram of acrylamide content in samples containing L-asparagine: C1, C2, C3 – control samples untreated TsA; S1, S2, S3 – samples after TsA treatment.

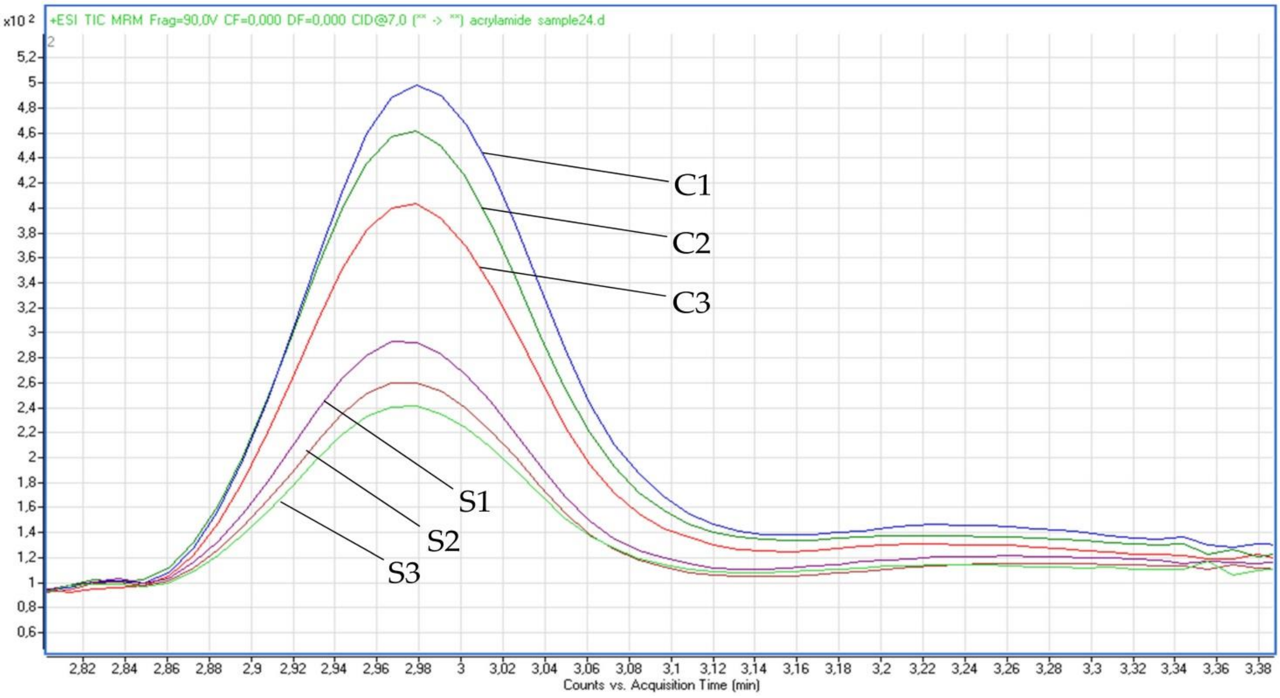

Figure S4. LC-MS/MS-chromatogram of acrylamide content in samples containing D-asparagine: C1, C2, C3 – control samples untreated TsA; S1, S2, S3 – samples after TsA treatment.
